# Microbial Fuel Cell Based Thermosensor for Robotic Applications

**DOI:** 10.3389/frobt.2021.558953

**Published:** 2021-10-05

**Authors:** John Greenman, Arjuna Mendis, Jiseon You, Iwona Gajda, Ian Horsfield, Ioannis Ieropoulos

**Affiliations:** ^1^ Bristol BioEnergy Centre, Bristol Robotics Laboratory, University of the West of England, Bristol, United Kingdom; ^2^ Department of Applied Sciences, University of the West of England, Bristol, United Kingdom; ^3^ Bristol Robotics Laboratory, University of the West of England, Bristol, United Kingdom

**Keywords:** bio-robotics, thermosensor, thermoreceptor, MFC, biosensors, thermotaxis

## Abstract

On the roadmap to building completely autonomous artificial bio-robots, all major aspects of robotic functions, namely, energy generation, processing, sensing, and actuation, need to be self-sustainable and function in the biological realm. Microbial Fuel Cells (MFCs) provide a platform technology for achieving this goal. In a series of experiments, we demonstrate that MFCs can be used as living, autonomous sensors in robotics. In this work, we focus on thermal sensing that is akin to thermoreceptors in mammalian entities. We therefore designed and tested an MFC-based thermosensor system for utilization within artificial bio-robots such as EcoBots. In open-loop sensor characterization, with a controlled load resistance and feed rate, the MFC thermoreceptor was able to detect stimuli of 1 min directed from a distance of 10 cm causing a temperature rise of ∼1°C at the thermoreceptor. The thermoreceptor responded to continuous stimuli with a minimum interval of 384 s. In a practical demonstration, a mobile robot was fitted with two artificial thermosensors, as environmental thermal detectors for thermotactic application, mimicking thermotaxis in biology. In closed-loop applications, continuous thermal stimuli were detected at a minimum time interval of 160 s, without the need for complete thermoreceptor recovery. This enabled the robot to detect thermal stimuli and steer away from a warmer thermal source within the rise of 1°C. We envision that the thermosensor can be used for future applications in robotics, including as a potential sensor mechanism for maintaining thermal homeostasis.

## Introduction

The work presented is part of a long-term strategy to create a fully energetically autonomous living bio-robotic organism, where all aspects of the robot, energy generation, processing, sensing, and actuation, may conform once integrated to a loosely defined hybrid definition of a living robot. When designing robots that are bio-hybrid as well as bioinspired, it should be noted that a precise scientific definition of life is elusive (Brown and Bhella, 2016). Living organisms require a degree of biochemical autonomy through metabolic activities (Dupré and O’Malley, 2009; Brown and Bhella, 2016) that produce molecules and energy needed for survival. This level of energy autonomy is essential to almost all levels of life. Thus, in creating a living robotic entity, cellular metabolism plays a vital role. In defining a living robot, the biological component is able to create the energy required for self-maintenance by the processes of metabolism. Allometric scaling relates size to function in animals (Kleiber, 1947) governed by metabolism. We therefore direct our work towards the construction of a meso-scale, robotic organism with a size somewhere between that of a mouse (e.g., *Mouse musculus*) and a rat (e.g., *Rattus rattus*). These mammalian species may provide useful inspiration for the purposes of allometric scaling and energy density comparisons. The work presented in this study refers to the initial development of thermosensors based on Microbial Fuel Cells and very much draws inspiration from natural organisms in terms of thermoreceptors, homeostasis, and thermoregulation found in animalia.

Microbial Fuel Cells (MFCs) are bio-electrochemical transducers that convert biochemical energy (primarily carbon energy) locked in organic biomass, directly into electricity (Potter, 1911; Bennetto, 1990). The generic MFC topology consists of two half-cells—the anode and cathode—separated by a semi-permeable membrane material. Following the colonization of the anode chamber by a bacterial community, this then becomes negatively charged and produces an electromotive force; in the presence of an oxidizing agent (usually, oxygen from air), the cathode becomes a counter half-cell, thus producing open-circuit voltage. Closing the circuit (usually by applying an appropriate resistive load) will allow the electrons to flow from the anode to the cathode, causing charge to be delivered, releasing the energy produced in the MFC. Altering the physicochemical conditions of the microbes will result in a change of electrode outputs as the microorganisms adjust to a new state.

In robotics, MFCs are the only type of energy generators capable of converting (or transforming) wet chemical substrates into electricity using biofilm-electrode cell metabolism. This power generation and the energy autonomy aspect was demonstrated by Ieropoulos et al. (2005) in the EcoBot line of robots, a new class of energy autonomous robots with the concept of artificial metabolism (Ieropoulos et al., 2003; Melhuish et al., 2006; Ieropoulos et al., 2010), in which the energy requirements for self-sustainability of the robot were met by the conversion of organic matter or “food” by means of a microbial (cellular) element of the energy generator called a Microbial Fuel Cell.

The EcoBot line of work was preceded by two other examples of artificial agents that paved the way towards autonomous robots; Slugbot (Kelly et al., 2000) showed how organic fuel in the form of slugs, could be successfully recognised by a sophisticated image processing systema and collected, but not utilised. Gastrobot (Wilkinson, 2000) on the other hand, employed bacterial metabolism inside an artificial stomach, to produce reducing power (digested effluent) that was pumped into chemical fuel cells, which were used to recharge the bank of batteries that was running the train-like robot.

In terms of biological organization (Mader et al., 2006), our aim is to create an artificial bio-hybrid organism. Each MFC or group of MFCs based on construction and manipulation of physical parameters can perform the functions as a biological cell unit, tissue, or organ, although at the robotic level of complexity, the “cell” refers to an individual MFC (not each individual microbe).

In order for a robot to be truly autonomous, a means must be provided for the evaluation of its internal state (Arkin, 1989; McFarland and Spier, 1997). Sensors measuring fuel, internal temperature, and kinetic forces (mass, flow, or movement) would be part of this system, allowing the robot to achieve homeostasis. In mammals, temperature is perceived by activation of thermoreceptors capturing a broad range of temperatures (Zhang, 2015). Thermosensation is essential for thermoregulation, which maintains thermal homeostasis. Moreover, thermoreception enables thermotaxis: thermotactic behavior dependent on temperature and is a common survival and a predatory utility feature in animals (Viswanath et al., 2003; Rosenzweig et al., 2005; Gracheva et al., 2010). Thermotaxis is present in the field of robotics in various forms and on a large range of robots, from mobile robots (Deegan et al., 2006) to heat-seeking missiles in the military (Kopp, 1982). Thermotaxis in artificial living systems (Kengyel et al., 2009) has been well studied.

In addition to the traditional use as power sources, MFCs have been reported to work as information processing units (Tsompanas et al., 2017), actuators (Philamore et al., 2013), and sensors (Santoro et al., 2017; Kumar et al., 2018). As sensors, MFCs are used either directly or indirectly (Ivars-Barceló et al., 2018). In indirect use, MFCs are used as a power source for powering conventional sensors of electromechanical or electrochemical forms. Early research includes sensor powering using sediment MFCs by Beyenal et al. (Donovan et al., 2008; Dewan et al., 2010); also, further research in these areas is reviewed by Ivars-Barceló et al. (2018). Direct use of MFCs as sensors utilizes the characteristic of power variation with a change of physicochemical conditions. Direct use of MFCs is commonly found in sensor application in the chemical domain, such as sensing COD or BOD (Cui et al., 2019).

In principle, the power output from MFCs changes with temperature; this is well described in the literature (Larrosa-Guerrero et al., 2010; Zhang et al., 2014; Ivars-Barceló et al., 2018). Hitherto, this phenomenon regarded as a parasitic effect was not considered as a sensory mechanism in robotics.

This paper, therefore, presents a custom designed MFC, which behaves as a thermal detector, whereby change in external temperature results in a change in output power. We explore the possibility of maintaining mean DC voltage using the external resistance and anolyte feed rate into the MFC as control variables. By using feedback control, we examine the sensors ability to maintain a fixed output voltage when not sensing, resulting in lower settling times in comparison to uncontrolled use.

In this work, we aim to describe the design and fabrication of small-scale MFC-based thermosensors (MFCTS), with MFCs acting as the self-sustainable thermoreceptors, akin to those found in Animalia. We examine thermoreceptor properties and exploit them in creating a closed-loop thermosensor. Finally, we apply the thermosensors in a robotic thermotaxis application. To the authors’ best knowledge, this is the first time an MFC-based thermal sensor is reported in a robotic application.

## Materials and Methods

This section describes the construction of MFCTS, MFC inoculation procedure, descriptions of the experiments for characterizing MFCTS, description of the robotic test platform, and the experiments conducted with the robotic hardware-in-the-loop (within the robot).

### Sensor Construction

The anodic assembly was primarily a carbon fiber tow (Hexcel Corp., United Kingdom) of 35 × 2 × 0.1 mm attached to a 316 stainless steel wire of 10 cm, ⌀0.3 mm current collector. The two elements were bonded by a 5 mm length of wire wrap, with the current collector at one end of the tow.

The cathode material was prepared by mixing activated carbon powder (G. Baldwins and Co., United Kingdom) and 20% PTFE (60% dispersion in H_2_O, Sigma Aldrich, United Kingdom) applied onto PTFE treated carbon veil sheet (Gajda et al., 2015). The cathode assembly consists of 20 × 10 × 0.4 mm cathode material, pressed against a 316 stainless steel plate of 20 × 10 × 0.4 dimension. A ⌀0.3 mm, 316-gauge stainless steel wire of 10 cm length was connected to the current collector plate by a wire wrap.

**FIGURE 1:**
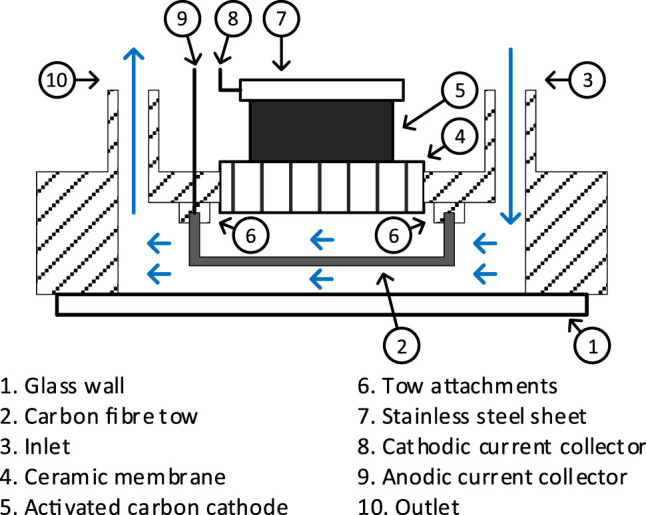
Schematic top view of the MFCTS assembly. Arrows depict the flow of anolyte through anodic chamber.

A top view of the assembly is illustrated in Figure 1. The sensor assembly primarily consists of a 50 × 32 × 10 mm, rapid prototyped ABS plastic hyper-rectangular chassis. The structure was designed to embed a single chamber MFC. The chassis features a diagonal liquid channel, which acts as the anodic chamber. Fluid (anolyte) inlet and outlet were located at the extreme ends of the liquid channel. The outer wall of the anodic chamber was a 40 × 22 × 0.2 mm rectangular thin glass wall, attached to the chassis using silicon adhesive (PN08027, 3M).

**FIGURE 2:**
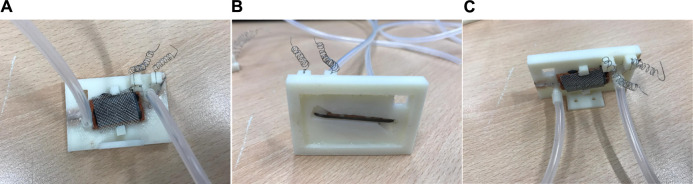
Photographs of the MFCTS unit. Back view **(A)**, front view **(B),** and top view **(C)**. Tubes carrying fluid shown connected to the unit [inlet on left and outlet on right in views **(A,C)**]. Output collected from current collector coils [cathode on left and anode on right in view **(A,C)**]. Front view (**B**) shows the primary surface including the carbon fiber tow exposed to the environment for sensing.

Within the anodic chamber, the ends of the anodic carbon fiber tow were mounted on two chassis points located at the furthest ends of the anodic chamber. The fiber tow was carefully positioned such that it enables contact with the free-flowing anolyte. The anodic material was placed in contact with the glass wall. A fired terracotta membrane of 25 × 10 × 1.5 mm separates the inner anodic chamber and the open-air-cathode assembly in the cathodic chamber. The ABS chassis was coated with butanol (Fisher Scientific, United Kingdom) for the reduction of liquid (ionic) conductivity between anode and cathode. Both anodic and cathodic current collector wire ends were coiled for the tension release. Figure 2 shows the completed sensor. Three identical replicates (MFCTS 1, MFCTS 2, and MFCTS 3) were assembled, inoculated, and operated in an identical manner.

### Experiment Setup

**FIGURE 3:**
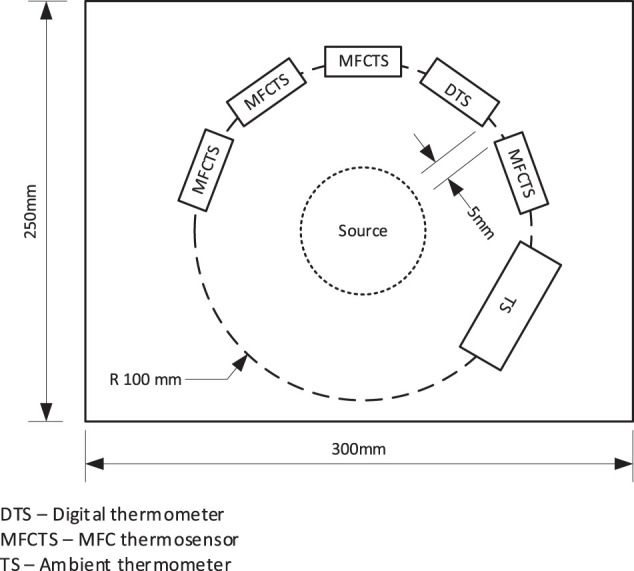
Arrangement of the MFCTS bench test setup. Multiple MFCTS and an electronic temperature sensor were fixed around a thermal source in a circular arrangement with a radius of 10 cm.

The laboratory test setup employed five sensor modules. The modules were mounted at 32.5°, 5 mm apart in a semi-circular pattern of radius 10 cm around a thermal source, which was placed in the center of the semi-circle (see Figure 3). Of the five sensor modules, four were MFCTSs and one was a structural dummy identical to the MFCTS but contained an electronic temperature sensor (Sensirion SHT31-DIS-B range: 0–90°C accuracy: ±0.2°C) with digital output. Controlled ambient laboratory temperature was set to 25°C. All MFCTSs were fed at a fixed flow rate of 73 µL/min in continuous mode using individual fluid transport. A single multichannel pump (Watson Marlow, United Kingdom) with tubing of bore diameter 2.05 mm was used for feed circulation. MFCTS data was sampled using a multichannel Agilent 34972A data logger. Digital temperature sensor provided direct output. Sample rates varied on individual experiments. A ∼30 mm candle flame from ∼10 mm wick of 6 mm diameter candle was used as the warm thermal source and a 50 mm diameter 250 ml beaker filled with ice was used as the cold thermal source.

### Inoculation

Four MFCTS cells were inoculated in a multistep approach: for a period of 96 h, cells were kept under batch fed conditions with anaerobic activated sludge (Wessex Water, Saltford, United Kingdom), enriched with 1% tryptone and 0.5% yeast extract (Winfield et al., 2014). After 96 h, the MFCs were continuously fed with sterile TYE (1% tryptone, 0.5% yeast extract in deionized water) at 73 µL/min. After a period of 8 days, load resistance of 10 kΩ was attached. After 21 days of experiment, the feed was changed to neat urine and left to stabilize. The ambient room temperature was set to 25°C, although the measured temperature oscillated between 23 and 24°C.

The following investigations were conducted in order to characterize the MFCTS:

1) External temperature variation effect on the MFCTS: warm and cold thermal sources were placed on the test rig, and data were recorded every second. 5- and 10-minute intervals were given for the MFCTS to stabilize. The responses were monitored and plotted. Flow rate maintained at 73 µl/min and external load maintained at 10 kΩ.
2) External temperature variation effect on the MFCTS under a variable load resistance: warm thermal sources were placed on the test rig. For a period of 60 s, and the output was observed. A time interval was given for the sensors to return to starting voltage. The flow rate was maintained at 73 µl/min. The external load was varied between experiments at 8, 10, and 12 kΩ. A minimal interval of 3 h was given between changes of load in order for the cells to stabilize.
3) External temperature variation effect on the MFCTS under a variable flow rate: warm thermal sources were placed on the test rig, for a period of 60 s, and the output was observed. A time interval was given for the sensors to return to starting voltage. The external load was maintained at 10 kΩ. The flow rate was varied between experiments at 73, 337, and 698 µl/min. A minimal interval of 3 h was given between changes of load in order for the cells to stabilize.
4) External temperature variation effect on the MFCTS with change in the duration of external temperature application.

### HIL Experiment Setup

**FIGURE 4:**
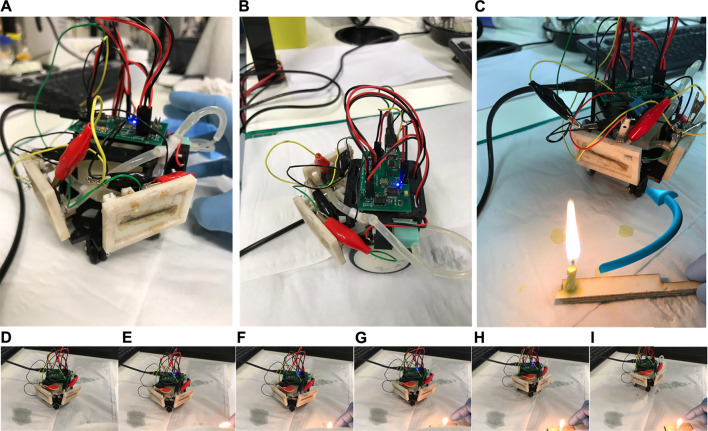
HIL test robot platform. Front view **(A)** with the MFCTS mounted diagonally on the front of the robot, side view **(B)** showing the electronic controller, pump, and fluid transport. **(C)** The robot in thermotaxis operation, moving away from the thermal source, making a circular turn as shown by the arrow in blue. Views **(D–I)** show the image sequence of the robot movement during thermotaxis operation after thermal source application in **(E)**.

The hardware-in-the-loop (HIL) testbed consisted primarily of a two-wheeled mobile robot platform (Figure 4). The platform facilitates control, data acquisition, and fluid management for the two MFCTSs. The physical structure provided mounting for MFCTSs, two motors for bi-directional movement, a pump for feeding the thermal sensors, a reservoir tank for holding feed for the MFCTS, and an electronic controller. The sensor modules were mounted at 100°, 5 mm apart. The electronic controller unit operated on 5 V, 500 mA and contained the following functions: data acquisition and signal processing, motor control, cell stabilizer, and feed regulator used for the application for robot thermotaxis. The data acquisition unit is capable of 16-bit analog to digital capture at 2 KSPS using a delta-sigma converter. The signal processing function managed signal conditioning, filtering, and post-processing. The motor control functionality enables motors for robot movement and controls feed pump functions. The cell stabilizer loop maintained the cells at a steady state in closed-loop control mode. In the active mode, the loop exerts external resistive loading from ∼300 Ω to 18 kΩ and manages continuous feeding of MFCTSs using the feed regulator. The feed regulator managed continuous feeding of MFCTSs in both open-loop and closed-loop modes, with feed rates of up to 500 µl/min. The onboard reservoir tank holds a fill volume of 40 ml; on normal operation, the fluid is recirculated back to the tank. The controller contained the application program for the overall robot management and robot thermotaxis.

The following application experiments were conducted using MFCTS mounted on the mobile robot (HIL):

1) Detection of external temperature increase: a thermal source was exposed perpendicular to each of the MFCTS, at a distance of 10 cm, for a time interval between 30 s and 1 min. The flow rate was maintained at ∼100 µl/min. The external load was maintained at 10 kΩ.
2) Closed-loop control: the closed-loop controller maintained a steady-state voltage of 30 mV. This was achieved by manipulation of the external load. The flow rate was maintained at ∼100 µl/min.
3) Detection with closed-loop control: a thermal source was exposed perpendicular to each of the MFCTS, at a distance of 10 cm, for a time interval between 30 s and 1 min. The flow rate was maintained at ∼100 µl/min.

In all experiments, the external data acquisition was recorded every second. The internal data acquisition device within the robot sampled at a determined frequency between 0.5 and 1 Hz; this was application-dependent.

## Results and Discussion

### Thermal Absorption

The construction of the sensor was focused on its ability to trap optimal heat transferred by radiation and convection. This was achieved by the selection of appropriate material and size/dimensions.

**FIGURE 5:**
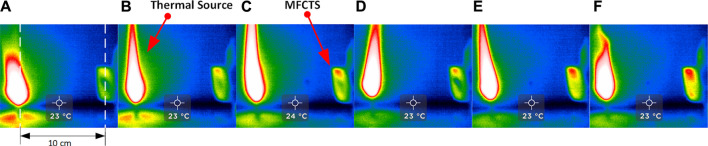
Thermal absorption of the MFCTS. The figure shows thermal imaging sequence [frames **(A–F)**] of MFCTS sensor, fixed 10 cm distance from a candle flame acting as the thermal source, with frames taken 30 s apart. It can be clearly seen that the MFCTS thermal signature changes from green **(A)** to red **(F)** as it absorbs thermal energy.

In order to optimize directional heat-trapping, the primary sensing surface was chosen to be infrared absorbent alkali-alkaline earth silicate type glass (conductivity: 1.06 W/m·°C, specific heat capacity: 0.84 J/gm K.), characterized by lower spectral transmittance characteristics at infrared wavelengths, provided better thermal radiation absorption in comparison to borosilicate glass (Schott, 2005; Karazi et al., 2017). A thinner glass plane (0.13 mm) provided a minimal difference in heat transfer and lower thermal capacitance promoted faster response. Figure 5 demonstrates the thermal absorption function of the glass surface. Images A to F were taken at 30 s intervals, using a thermal camera (Seek thermal imaging, UPC: 855753005655); a candle was placed 10 cm apart, perpendicular to the sensing surface of the MFCTS. It was visible that the glass surface became warmer faster in contrast to the rest of the sensor assembly body.

The diagonal orientation of the fluid channel containing the linear anodic chamber restricted and lessened the air trapping within the chamber. The chamber was kept completely filled to minimize air in the chamber, in order to maximize generated open-circuit voltage via anaerobic microbial metabolism (Winfield et al., 2019). The anodic chamber had a volume of 0.22 ml, in order to promote a lesser effect of the continuous anolyte feed on the sensing surface. As illustrated in Figure 5, as water at room temperature (simulating anolyte feed) was pumped into the fluidic channel at a flow rate of 73 µl/min, it was visible that the diagonal fluid channel across the MFCTS appeared to be the last portion of the sensing surface to thermally saturate. The linear tubular shape of the channel promoted laminar flow and reduced viscous drag. The MFCTS had a mean hydraulic retention time of ∼3 min at 73 µl/min.

The thermal stimuli affected both sensors, but the nearest and the parallel sensor absorbed more energy than the furthest. The voltage differences of each pair is relative to prior stimulation DC voltage. This is clearly visible in Figure 11. ***B***: {0.182, 0.66}, {0.1, 0.26}, {0.53, 0.349}, {0.24, 0.292}, {0.318, 0.063}, {0.06, 0.225}, {0.444, 0.011} mV.

### MFCTS Characterization

MFCs are classically classified as current generators; hence, it is conventional to analyze MFC systems in terms of power. For application purposes, however, it is simpler in electronics to capture/work with voltage signals. Thus, the characterization emphasizes on voltage signals.

#### Preliminary Behavior

**FIGURE 6:**
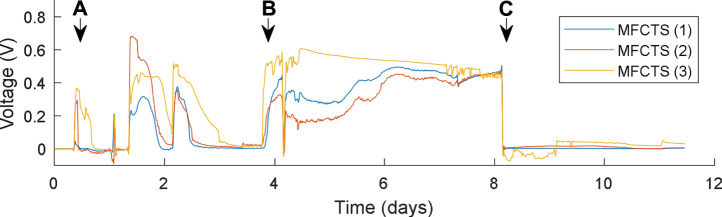
Biofilm inoculation process. MFCTS were batch fed from point **(A)**, until point **(B)**, after which continuous feed commences. Resistive load connected at point **(C)**.

For the primary component of the MFCTS being the MFC, a biofilm was introduced to the MFCTS anode. The process is illustrated in Figure 6. From ***a*** till ***b,*** the inoculum was fed in batch mode at a very low flow rate of 8 µl/min, in order to discourage clogging of the fluidic channel. After day 4, TYE was fed continuously at 73 µl/min, this being the slowest, yet the most effective flow rate the pump could accommodate for prolonged operation. A fixed resistive load of 10 kΩ was added on day 8 ***c***, at which an open-circuit value of 0.466 V was lowered to a stable mean voltage of 20 mV, 24 h after the load was attached. MFCTS (2) and (3) were lower in voltage, compared to MFCTS (1). Cells were left to stabilize for 7 days, after which a stable mean voltage of 30 ± 3 mV was attained before commencing with the experiments. Of the batch of three tested identical MFCTS, MFCTS (3) was comparatively noisy, whereas MFCTS (1) and (2) were more stable. Air bubbles were observed inside the anodic chamber of MFCTS (3) in constant feed flow. The default operating load resistor for the MFCTS was chosen such that the closed-looped voltage remained sustained between 25 and 35 mV.

#### In Relation to Flow Rate

**FIGURE 7:**
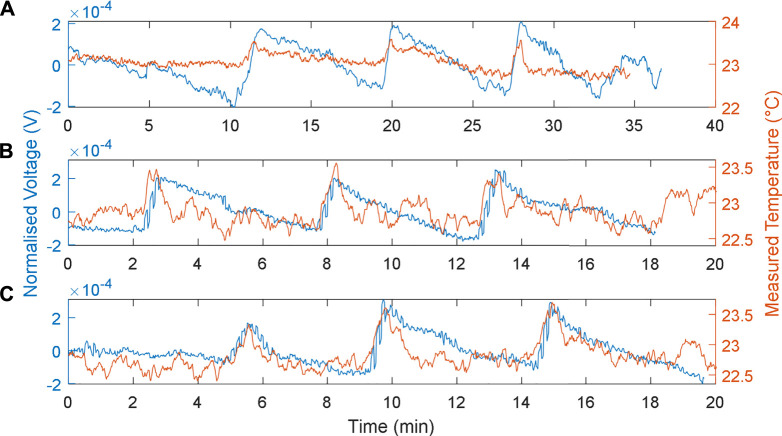
MFCTS sensor, with the change in flow rate. **(A)**: 73 µl/min, **(B)**: 337 µl/min, and **(C)**: 698 µl/min flow rates, with 10 kΩ load resistance. Brown trace: MFCTS; blue trace: electronic sensor.

The responses of the MFCTS with respect to the change of flow rates are depicted in Figure 7. The plots were normalized (V=v/||v||)  and detrended at mean DC voltage for analysis. Plot ***A*** depicts the default flow rate of 73 µl/min, with a normalization vector of (0.6536, 1.3591, 1.3752). The voltages were detrended at a DC mean 24 mV, with a slope of −1.91e-5. Plot ***B*** depicts a flow rate of 337 µl/min, with a normalization vector of (0.4750, 0.9890, 0.9874). The voltages were detrended at a DC mean of 24.7 mV, with a slope of −4.37e-6. Finally, plot ***C*** presents a flow rate of 698 µl/min, with a normalization vector of (0.5132, 1.0892, 1.0815), with the voltages detrended at DC mean 26.1 mV at a slope of 1.39e-5. The MFCTS were analyzed in terms of produced power through the metabolism process; plots depict voltage response at a fixed resistance of 10 kΩ. Figure 7 also presents the reference temperature measurements using the electronic sensor.

As expected (Walter et al., 2016) as the flow rate was increased, the DC mean power increased for MFCs. This was also true for the MFCTS as can be seen from Figure 7: for the flow rates 73, 337, and 698 µl/min at loading, the mean voltage vector was (24, 24.7, 26.1) mV. In contrast, a rise in power due to the thermal activity was not visible. Applying a heat source at different flow rates did not render a higher output power. Thermal power gain (in terms of voltage at 10 kΩ) over the change in flow rates resulted in voltages of 0.526 ± 0.266, 0.341 ± 0.107, 0.317 ± 0.125 mV (SD). This was most probably attributed to the transfer of thermal energy away from the MFCTS by the fluid flow in and out of the MFCTS.

As flow rates increased, it was observed that the rise times (10–90%) decreased. The rise times for the plots were calculated as 39.11 ± 10.18, 28.35 ± 5.21, and 25.95 ± 3.62 s (SD). It was deduced that the increase in flow rate contributed to the increase in power, but this was compensated by the flowing fluid capturing the thermal energy. It was observed that, with the increase in flow rates, the fall times decreased. The fall times (10–90%) for each of the flow rates were calculated to be 316.53 ± 10.86, 222.13 ± 12.40, and 171.47 ± 9.17 s (SD). The decrease in fall times increased the responsivity of the MFCTS. This was attributed to the thermal mass of the anolyte transporting thermal energy in and out of the system at a higher frequency, causing the sensor to reach thermal equilibrium faster. This was clearly observed in the overall pulse periods, as it steadily decreased for the increase in flow rate. The pulse periods were calculated as 8.17 ± 0.29, 5.23 ± 0.31, and 4.67 ± 0.62 min (SD).

During standard operating conditions, the porosity of the ceramic material could allow partial oxygen to penetrate towards the anode electrode resulting in a lowered open-circuit voltage as oxygen in the chamber contribute to a more positive anode redox potential (Winfield et al., 2013; Winfield et al., 2019). At higher flow rates in the range of 698 µl/min ∼1.395 ml/min, air bubbles were visible in the anodic chamber. This phenomenon was attributed to the Bernoulli drag causing the porosity of the ceramic material to draw air from the external environment.

It was important to have the MFCTS fed continuously, as we wished to maintain the MFCTS in a steady state and keep physiochemical properties constant (Ledezma et al., 2012; Oliveira et al., 2013).

#### In Relation to Load Resistance

**FIGURE 8:**
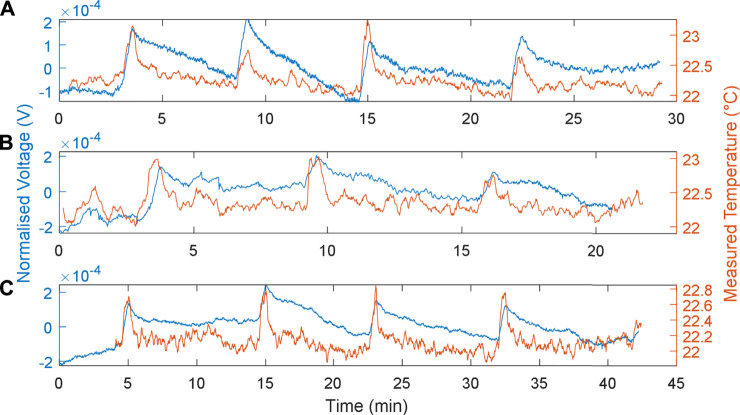
MFCTS sensor with the change of load resistance. **(A)**: 8 kΩ, **(B)**: 10 kΩ, and **(C)**: 12 kΩ, at 73 µl/min flow rate. 1 min of thermal source exposure. Brown trace: MFCTS; blue trace: electronic sensor.

The responses of the MFCTS with respect to the change of load resistances at a fixed flow rate of 73 µl/min, at 1 min of thermal source exposure, are depicted in Figure 8. The plots were normalized (V=v/||v||)  and detrended at a mean DC voltage for analysis. Plot ***A*** depicts the load resistance of 8 kΩ, with a normalization vector of (0.379, 0.883, 0.023). The voltages were detrended at a DC mean 18.2 mV, with a slope of −3.26e-5. Plot B depicts the default load resistance of 10 kΩ, with a normalization vector of (0.4750, 0.9890, 0.9874). The voltages were detrended at a DC mean 21.9 mV. Finally, plot ***C*** presents the load resistance of 12 kΩ, with a normalization vector of (0.05, 0.645, 1.468), with the voltages detrended at DC mean 25.3 mV at a slope of 1.08e-6. As previously, the MFCTS were analyzed in terms of produced power through the metabolism process; the reference temperature measurements using the electronic sensor were also presented.

The resistive values 8, 10, and 12 kΩ were chosen such that the highest load was able to sustain the MFCTS longer than 3 h at a positive gradient of DC mean. As the resistive load decreased in the order 8, 10, and 12 kΩ, the DC mean voltage was seen to increase; the mean DC values are 18.2 ± 0.23, 21.9 ± 0.11, and 25.3 ± 0.17 mV (SD). The power output was calculated as 41.40, 47.96, and 53.34 nW, indicating that the MFCTS as MFCs were operating in the left-hand side of the polarization curve; the power output was increased as the resistive load was reduced. A decrease in load resistance also caused the rise time to decrease by ∼1 s. The rise times (10–90%) were calculated as 33.07 ± 2.81, 32.27 ± 2.64, and 31.73 ± 2.30 s (SD). In contrast, the fall times (10–90%): 270.93 ± 3.24, 274 ± 1.1314, and 361.87 ± 29.98 s (SD), increased significantly as the resistances increased. The sharpness of the responses decreased as resistance was increased. The temperature difference vector for plots ***A***, ***B***, and ***C*** is (1.34, 1.03, 1.00)°C; the decrease in load resistance resulted in a miniscule thermal gain. The gains were given as 0.15 ± 0.05, 0.19 ± 0.06, and 0.22 ± 0.08 mV (SD).

#### In Relation to Stimulus Duration

**FIGURE 9:**
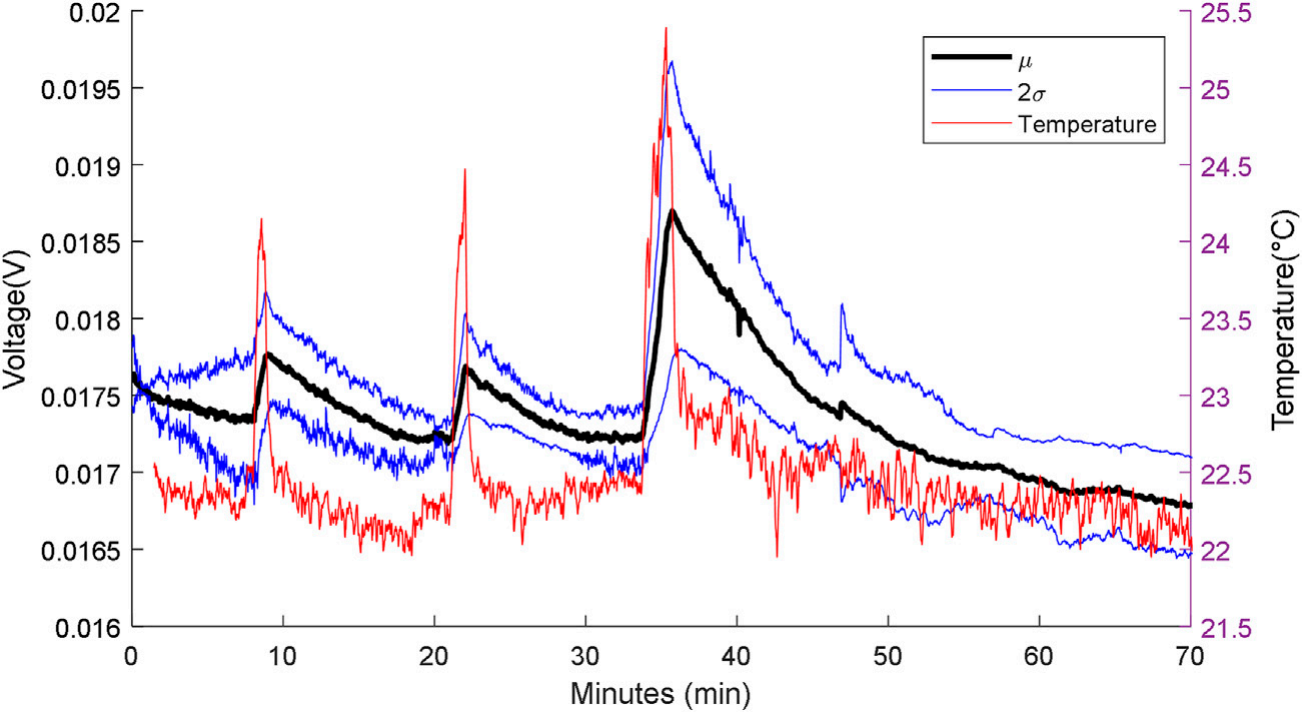
MFC sensor w.r.t varying heat source stimuli periods. From left to right: first peak: 1 min, second peak: 2 min, and the third peak: 3 min. Flow rate 73 µl/min, with 10 kΩ load resistance.

The responses of the MFCTS sensor with respect to varying stimulus periods are depicted in Figure 9. The plot shows the normalized mean DC power output response (voltage signal at 10 kΩ), with a normalization vector of (3.1226, 8.2515, 6.4316), standard deviation window (2σ), and the measured reference temperature. The flow rate was kept constant at 73 µl/min. Peaks from left to the right of the plot correspond to stimulus times of 1, 2, and 3 min.

At a steady state, the MFCTS maintained a mean DC voltage of 24.0 ± 7.9 mV (SD). For a measured thermal difference of (1.88, 2.31, 3.03) *°C* for the three pulse stimuli, the thermal gain increased in power as the pulse stimuli period increased. The gain was measured to be 0.61 ± 0.13, 0.65 ± 0.17, and 1.95 ± 0.47 mV (SD).

The rise and the fall times of the MFCTS resulting from the thermal stimulus were compared to the curve trend of the electronic sensor. The rise times (10–90%) of the MFCTS, 45.07 ± 3.03, 52 ± 5.77, and 98.93 ± 2.57 s (SD), increased following the trend of the electronic sensor (10–90%) (27.52, 47.20, 77.60) s. Similarly, the fall times (10–90%) of the MFCTS, 419.20 ± 28.48, 412.53 ± 95.94, 908.53 ± 87.22 s SD, increased similar to that of the electronic sensor (10–90%) (42.48, 83.20, 95.20) s, but with higher magnitude.

Increasing the stimulus period caused a higher magnitude of thermal energy absorption resulting in a temperature rise. The sensor also acted as a thermal buffer, which stored the captured energy for longer periods, hence, the longer fall time. Thus, increasing the stimulus period resulting in longer exposure to the stimulus required a higher time interval for the sensor to return to its default state.

From these results, it was visible that, when using the MFCTS for an application such as thermotaxis, it was easier to move away from the source than to go towards it, as moving towards the source will result in requiring further time to dissipate the energy and recover to the pre stimuli state.

### MFCTS for Thermotaxis Application

Thermotaxis provides behavioral thermoregulation mechanisms for allostasis (McEwen and Wingfield, 2003) and also supports predatory, survival function in nature. In this section, two MFCTS based thermosensors were installed on HIL mobile robot platform to detect the rise in temperature from a directional source and then turn away from it.

For the thermotaxis application, the thermosensor control mechanism needs to keep track of the state of the sensor; this includes DC mean voltage and the active system state. The thermotaxis application was tested on a HIL robot platform. Within the robot, power generated by each MFCTS was converted to a digital voltage signal, with an analog to digital converter at an effective resolution of 15 bits, 62.5 µV per step, at a sampling frequency of ∼8.2 Hz. A fixed load resistance of 10 kΩ was used for voltage output. The captured signal was then processed through a filtering operation. This was primarily due to limitations in the quality and characteristics of the electronic system within the HIL platform. A one-dimensional Kalman filter (KF) (Mendis, 2015) with (Q = 0.01, *R* = 0.1) was used in order to eliminate noise and other quantization errors. Alternatively, a band-pass filter could be substituted for the application. A KF was used here due to the robustness and ease of software implementation. The filter requires time to stabilize after initialization as the causality of the KF causes an offset.

With the objective of keeping the complexity of the thermal stimuli detection mechanism to a minimum, a scheme based on the rate of change of voltage of MFCTS (dV/dt) was chosen for the stimuli detection. The sampled voltage signal from the MFCTS was down-sampled to ∼0.163 Hz for creating a slope window of length 50. A value of 10^–5^ was set heuristically as the triggering threshold for slope values to be considered as peaks. Valid stimuli were then chosen by rejecting peaks less than a time window of 3 samples, thus resulting in 18 s lag before issuing commands for the robot to move away from the source.

**FIGURE 10:**
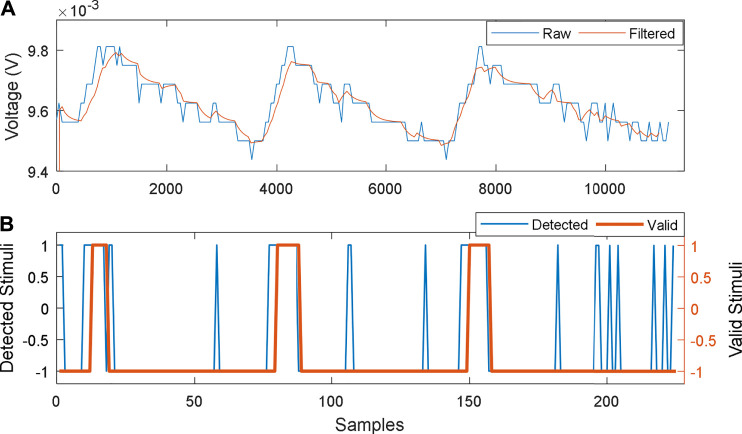
MFCTS stimuli detector process. **(A)**: raw output of MFCTS and filtered output. **(B)**: output of the peak detector: all detected peaks and valid detections. Samples corresponding to **(A)** and **(B)** are 0.122 and 6.1 s.

The detection process using the HIL test robot is depicted in Figure 10, in which 3, thermal stimuli were directed from a distance of 10 cm, for a period of 1 min. Each stimulus was detected, as valid peak after a period of 18 s minimal interval between stimuli was 384 s. The sensor reverted to the previous DC mean of 0.244 ± 0.02e-2 mV (SD), after 318 ± 25.5 s (SD). The achieved thermal gain for each stimulus was 9.6 ± 9e-2 mV.

MFCs with identical physiochemical conditions tend to provide responses with similar trends during steady-state operation; however, due to slight differences in construction, biofilm maturation, stress, and stimuli, similar MFCs provide mild differences in power output characteristics. MFCTS inherit this; thus, no two MFCTS provide identical output to stimuli in open-loop operation. These result in DC mean voltage variations between MFCTS and within MFCTS intermittently after stimuli. This was averted using a feedback control system, where the DC mean output was regulated.

For the feedback controller, a first-order system model can be approximated from data of Figure 6 point ***c.*** Assuming connecting a load resistor to the open circuit of MFCTS as a load, the system parameters were found to be K = 0.0457 and τ=−0.1 .

A proportional controller, with a gain of 0.7 was used for regulating the output voltage of the MFCTS, as it needed to be dampened in order to prevent overshoot as the biofilm in the MFCTS, which may be overstressed. DC mean was set as a reference voltage to a value of 8 mV. The control loop activates every 300 s. The signal capture and detection process was identical to the previous but with an ADC sampling frequency of 1 Hz and the detection mechanism down-sampled the signal to 0.17 Hz.

**FIGURE 11:**
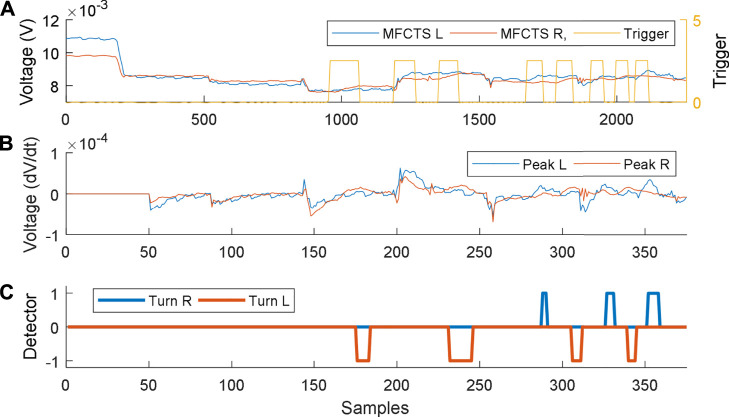
Closed-loop control of MFCTS, within HIL. **(A)**. Two MFCTSs with output voltage reference at 8 mV. Application of a thermal source acts as a trigger. **(B)**. Peak detection algorithm detects all peaks above the threshold. **(C)**. Robot commands resulting from peak detection. Samples correspond to 1 s **(A)** and 6 s **(B)**.

The respective plots for the closed-loop controller in operation are shown in Figure 11. The controller operates every 300 s, to stabilize the system to the reference DC mean voltage of 8 mV. Stimuli of 60 s were applied between minimum time intervals of 160 s (plot ***A***). Plot ***B*** illustrates the slope of voltage variation. The thermosensory system requires 18 s to detect a valid pulse. The maximum time taken to stabilize was 300 s (plot ***C***), which was equal to the time period of the control loop activation. Once the signals were detected, the robotic controller commanded the mobile robot to turn 90° direction opposing the direction of stimulus and move for 3 s (corresponding to ∼5 cm). This resulted in faster settling of the MFCTS resulting in the ability to accept stimuli faster than the open-loop system.

### Functional Comparison

The functional concept of the artificial thermosensory system (ATS) incorporating thermoreceptor MFCTS and the surrounding ecosystem is akin to that of mammals and could be explained in terms of the human thermosensory system (HTS), as it is well studied.

Topologically, the ATS consists of MFCTS, signal conduction, detection, and processing. Given adequate stimuli, the MFCTS responds as a power signal, intercepted and processed using an electronic microprocessor to bring about perception. Similarly, the HTS consists of the sense organ, sensory receptors, and the pathway to the central nervous system (CNS). Sensory receptors respond to an adequate stimulus. Within the CNS, the sensory information is processed to bring about perception (Rhoades and Bell, 2012). The MFCTS convert organic matter to power, thus producing an aperiodic power signal. The power signal varies in amplitude during sensor stimulation. Electrical connections transmit these signals and electronics process them after filtering and applying conventional silicon electronics methods. In the context of this paper, the signals are processed using digital and embedded electronics in which a microprocessor gives perception. In comparison in the HTS, dedicated channels (labeled lines) for warmth, cold receptors, and nociceptors are preserved in spinal, thalamic, and cortical neurons (Schepers and Ringkamp, 2009).

Placement and the referencing of the ATS are application dependent: in thermotaxis application, sensors positioned at the external interface between the artificial organism and environment allow the robot to detect changes in the external environment relative to self-reference. In situations where differential monitoring between internal and the external environments are required, such as in the case of thermal homeostasis application, internal and external sensors are used in combination, with an insulated internal sensor with minimal thermal changes or any other physiochemical changes occurring; then, such an insulated sensor could be used as a reference output. Similarly, the HTS uses interoreceptors and exteroreceptors, internally and externally to the body. As a complex system, multiple sensor populations exist in multiple locations, with multiple reference temperatures (Houdas and Ring, 2013) primarily from the hypothalamus (Kenny and Flouris, 2014). Differentials between internal and external sensors enable feed forward control and actions from the thermoregulatory system to prevent damage to the organism as a whole.

In the HTS, external sensations of warmth and cold mediate through dedicated thermoreceptors, embedded in the membranes of afferent fiber terminals as free nerve endings in the skin. In addition to thermoreceptors, noxious heat (above 40°C) and noxious cold (below 17°C) (Pongs, 2009; Schepers and Ringkamp, 2009) are fused (sensor fusion) with nociceptors—the receptors for pain stimuli. This is also true with regard to the ATS. Using multiple sensor populations within the same region increases detection of the overall accuracy of the characteristic of the thermal change. Individual MFCTSs vary mildly by construction and in their dynamic steady states.

In both systems, the general transport mechanism involves translating thermal energy into electronic signals. In the HTS, the mechanism of thermal transduction is understood to involve the temperature dependent action of transient receptor potential (TRP) ion channels (Schepers and Ringkamp, 2009). Three of the ion channel groups make up the key thermoreceptors. Each thermoreceptor operates over a specific temperature range, thereby providing a potential molecular basis for peripheral thermosensation. Activation of ion channels is caused by ionic channels Na/K transport mechanism (Rhoades and Bell, 2012). Activation of all thermo-TRP channels results in an inward, nonselective cationic current and consequent depolarization of the resting membrane potential (Rhoades and Bell, 2012). The receptor potential is also a gradient response depending on the stimulus intensity; this is similar to the response seen in Figure 9, in which the thermal gain is proportional to the stimulation intensity.

For a stable and constant DC power output of an MFC aspect of the MFCTS, the microbial element needs a stable physiochemical environment for steady-state operation. The kinetic and mass transfer processes of the MFC system are bounded by parameters such as the activation energy, mass transfer coefficient, and solution conductivity. It is shown that changes in temperature significantly influence these processes (Gonzalez del Campo et al., 2013; Ivars-Barceló et al., 2018). Not only power but other properties, such as COD removal, changed with temperature (Pham et al., 2006; Jadhav and Ghangrekar, 2009; Vázquez-Larios et al., 2010; Oliveira et al., 2013). The increase of power density with an increase of temperature is also related to the enhancement of the microbial metabolism and membrane permeability and to the ohmic resistance reduction due to the higher conductivity of the liquid solution (Oliveira et al., 2013; Pietrelli et al., 2019). Thus, the output power signal from the MFC reflects the thermal changes in temperature within the MFC.

Although individual thermo-TRP channels are activated within a relatively narrow temperature range, collectively, their range is quite broad, from noxious cold to noxious heat (Schepers and Ringkamp, 2009; Zhang, 2015). In ATS, it is known that different types of microbial species respond/are sensitive to different temperature ranges. The influence of the temperature will change depending on the specific nature of the microorganism and the feedstock employed (Pietrelli et al., 2019). Different species will have different optimum temperatures (Oliveira et al., 2013).

ATS uses a feedback control mechanism periodically to maintain the MFCTS DC mean output; similarly, in nature, receptors saturate and inhibitors are required to silence channels to bring them back to normal (Noël et al., 2009). One method in avoiding saturation (Schepers and Ringkamp, 2009) is to have different ranges and redundancy.

### System Limitations

Similar to a biological organ, the MFCTS require continuous nutrient flow for operation. However, it is possible to provide the nutrients in periodically pulsed feed for operation. The effects of periodic pulsed feed need compensation at the control module. These effects and compensation requirements are intended for future study.

Stability is a key issue considering the MFCTS; the MFCTSs were required to operate in a dynamic steady state in order to maintain stability. Stability of the MFCTS affects the time to recover from stimulus. The use of multiple MFCTS increases the overall stability of the system and decreases the time to recovery and accuracy of the overall system.

The thermal absorption levels of the sensing surface limited the performance of the MFCTS by increasing the time to absorb thermal energy, as visible in Figures 7–9. The detection capabilities could be enhanced by increasing the absorption of the IR range, by replacing the glass interface with specialist glasses such as ionically colored absorbers (Schott AG, 2015).

MFCTS signals operate in the range of 10–50 mV, which are susceptible to electrical noise. This proves challenges because of the two dissimilar domains of biological and silicon electronics. Similar problems occur when interfacing electronics with human bio-potentials, which are usually in the range of 10 µV–100 mV.

Unlike the biological equivalent, the threshold detection component is separate from the MFCTS; future work aims at incorporating local threshold detection within the MFCTS. This allows less strain on the central detection mechanism.

## Conclusions

In the design of artificial bio-robots, MFCs provide a platform technology that exploits microbial metabolism. The living aspect of the robotics was defined by the aspect of energy generation from microbial metabolism. Each MFC or group of MFCs can be manipulated by design and construction to emulate a living cell, tissue or organ of biological form. In this work, we demonstrated the possibility of detecting external temperature changes using an artificial biological thermoreceptor within an artificial thermosensory system.

The thermoreceptor utilized the thermal properties of MFCs and was powered by microbial metabolism. The functionality of the MFCTS and the ecosystems producing the overall thermosensor were similar in terms of functional blocks to that of mammals.

The glass based sensory element of the MFCTS, shape, and dimensions of the anodic chamber and the material selection played a critical role in shaping the MFCTS responses. Characterization of MFCTS was performed under various external load conditions, feed regimes, and application purposes whereas the MFCTS responded to an adequate stimulus and converted the response to a power signal. As flow rate increased, rise and fall times decreased, indicating that fluid feed was able to capture some of the thermal energy and dissipate it out of the MFCTS. Characterization directed for application purposes (such as thermotaxis) showed that lower exposure was favorable for faster settling. Hence, in the practical application of robot thermotaxis, moving away from the thermal source has a faster settling time than towards the thermal source.

In evaluation of thermal stimuli detection, two thermosensors installed on a mobile robot platform were able to detect stimuli of 1 min after an 18 s time period of application, with a minimum of 318 s recovery time. The long recovery times were reduced to a maximum of 300 s by using a closed-loop controller with a proportional gain in order to stabilize the thermosensor. With the use of the controller, the robot was able to achieve thermotaxis application from stimuli every 160 s, without the need for complete MFCTS recovery, enabling the robot to detect thermal stimuli and steer away from a warmer thermal source within the rise of 1°C within the MFCTS.

## Data Availability

The original contributions presented in the study are included in the article/Supplementary Material; further inquiries can be directed to the corresponding author.
